# P-145. Parasitological characterization of stool samples from indigenous Wayuu children from La Guajira, Colombia

**DOI:** 10.1093/ofid/ofae631.350

**Published:** 2025-01-29

**Authors:** Derly Carolina Hernandez, Clara Judith Benavides, Angie Tatiana Saenz, Maria Valentina Sanchez Gil, Diego Sebastian Ariza Marín, Tatiana Alejandra Hernandez Diaz

**Affiliations:** HOSPITAL MILITAR CENTRAL, CAJICA, Cundinamarca, Colombia; Universidad Militar Nueva Granada, Bogota, Distrito Capital de Bogota, Colombia; Universidad Militar Nueva Granada, Bogota, Distrito Capital de Bogota, Colombia; Universidad Militar Nueva Granada, Bogota, Distrito Capital de Bogota, Colombia; Universidad Militar Nueva Granada, Bogota, Distrito Capital de Bogota, Colombia; Universidad Militar Nueva Granada, Bogota, Distrito Capital de Bogota, Colombia

## Abstract

**Background:**

La Guajira, 396,511 children and most of them recognized as Wayuu indigenous population. They have many public health problems in primary services. This children are at high risk of suffering from intestinal parasitosis. The most common are *Entamoeba histolytica* (EH) and *Giardia duodenalis* (GD) in their infectious forms. The common source is contaminated food and water. Geohelminth (GH) infections can be complicated by pulmonary compromise and worsening health of children with nutritional problems. In Colombia, primary care for intestinal parasitosis is albendazole. As part of the social interest we want to know the main species that we can find in the feces of a group of these children to know if primary treatment with albendazole is sufficient.

Coccidial structures by Ziehl-Neelsen (ZN)

**Figure:**
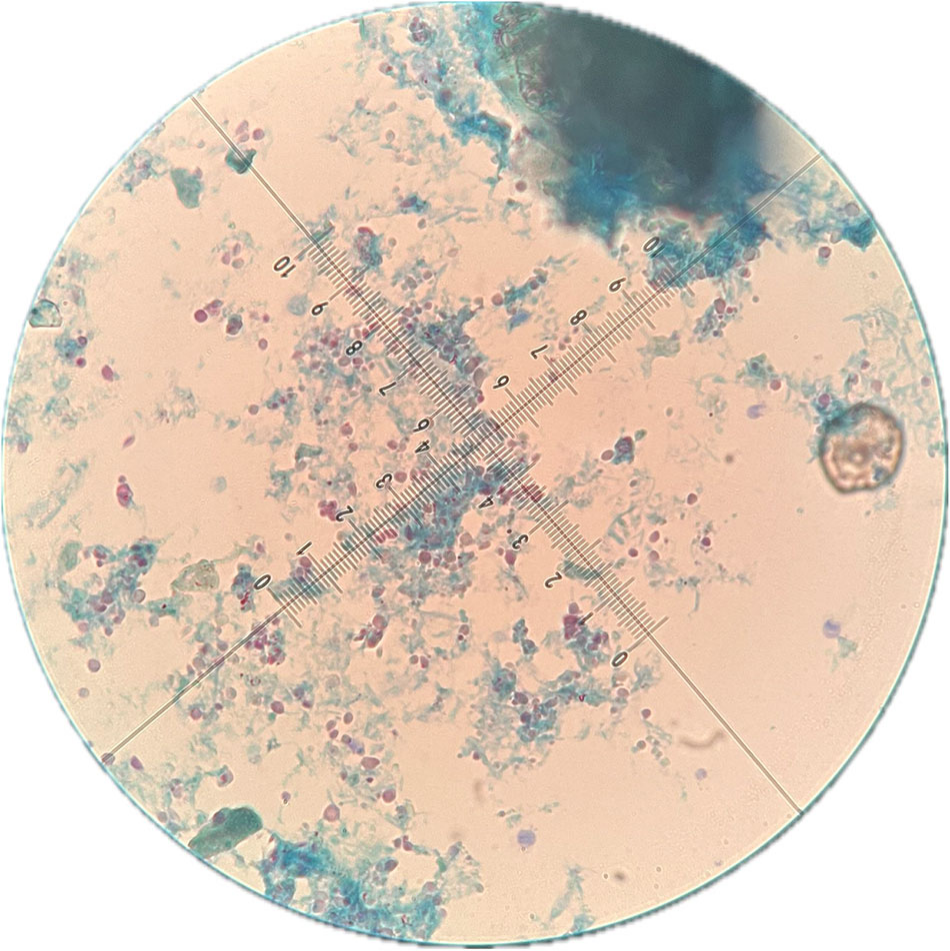

**Methods:**

Descriptive cross-sectional study. On December 2023 was assessing 44 coprological samples. Containers were labelled, consent and informed assent were signed. They were preserved in MIF reagent and stored at 4°C, processed by Formalin-Gasoline (F-G) with the addition of parasitological lugol for microscopic evaluation under 10x and 40x objectives. Coccidial structures were observed by Ziehl-Neelsen (ZN) staining with a 100x objective, both calibrated with a micrometer. The variables was analized by frequencies and measures of central tendency.

Trophozoite of Entamoeba histolytica

**Figure:**
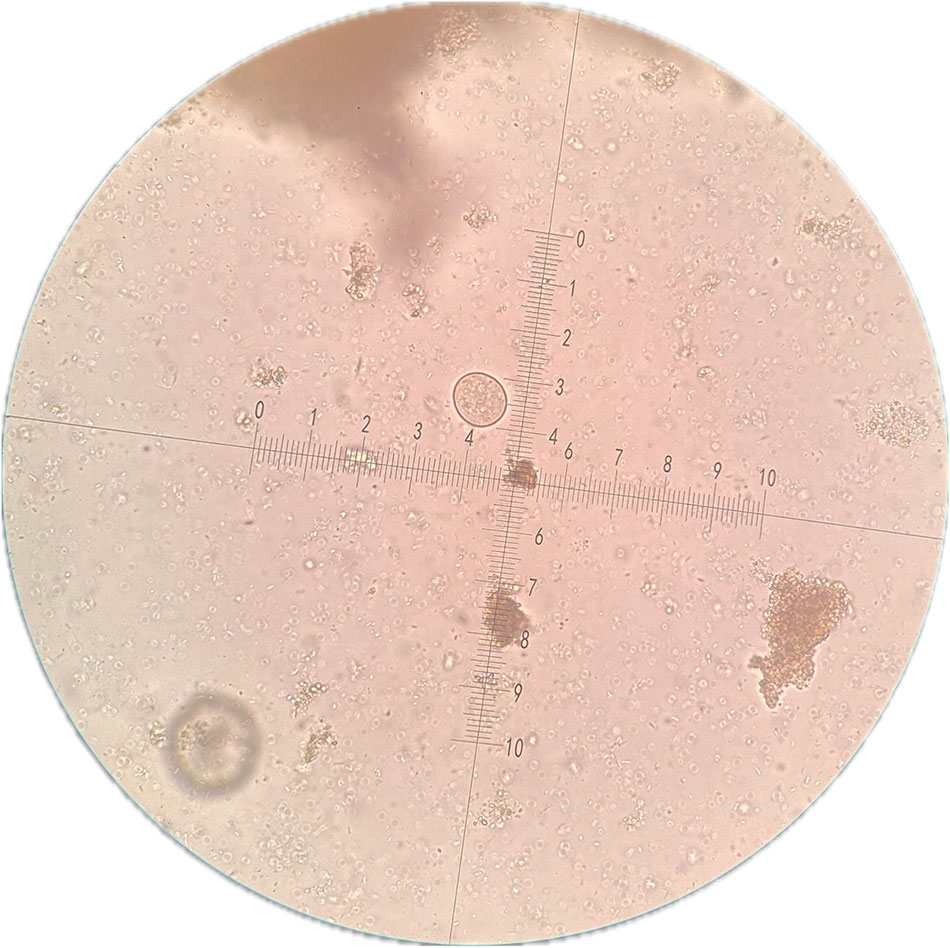

**Results:**

44 samples were included. Abnormal results were found in 42/44 (95.5%) by ZN technique; 44 (95.5%) *Microsporidium* and 23 (52.3%) *Cryptosporidium*. In the F-G and addition of parasitological lugol, abnormal results were found in 38/44 (86.4%). Regarding Protozoa, 18 (40.9%) were found GD cysts, 36 (81.8%) *B. hominis*, 8 (18.2%) *E. nana*, 12 (27.3%) EH trophozoites and 2 (4.5%) *C. Mesnili*. Regarding GH, 2 (4.5%) were found with *S. stercoralis* and *A. lumbricoides* and 1 (2.3%) *H. nana*. Both ZN staining and parasitological lugol staining showed 19 (43.2%)*C. cayetanensis* and 14 (31.9%) *C. belli*.

Cysts of Giardia Duodenalis

**Figure:**
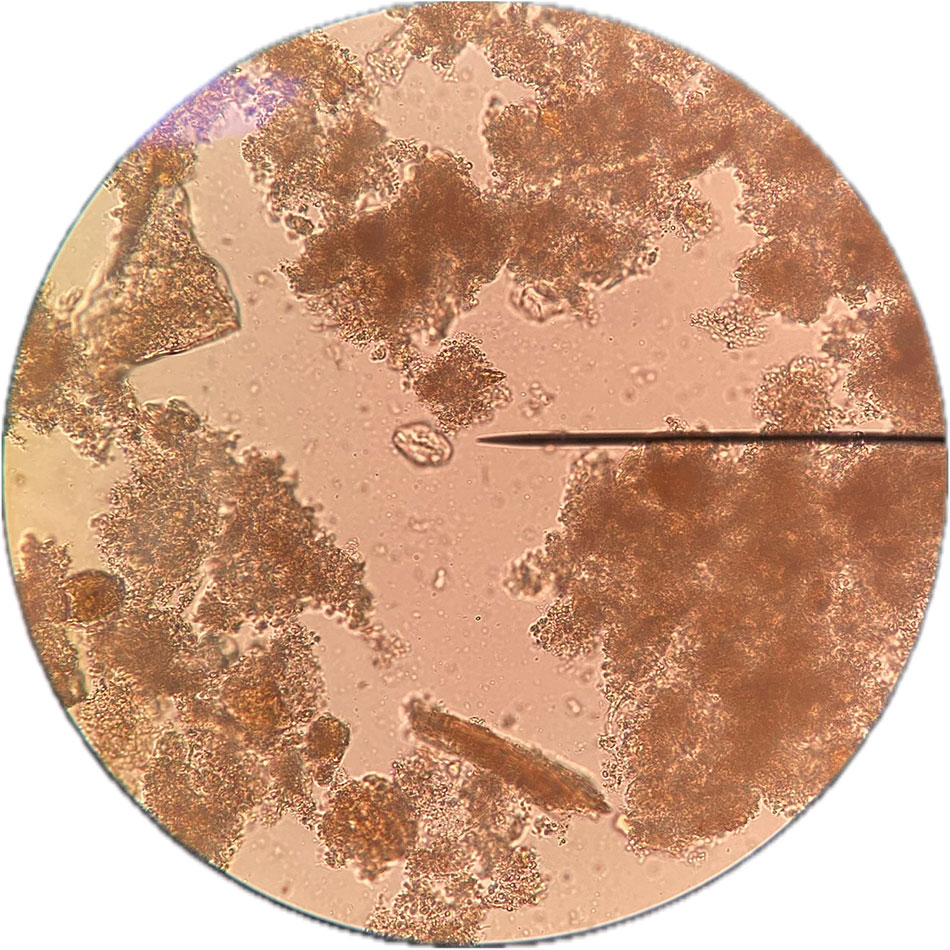

**Conclusion:**

The frequency of PI was 95.5%, 42 (95.5%) protozoan and 5 (11.4%) GH. The most frequent isolates were *Microsporidium*, *B. hominis* and GD. Perhaps we could offer another deworming treatment in this population. This description could be the basis for other studies in this population to offer better alternatives and make the public health situation in this scenario more visible to the government.

Egg of Hymenolepis nana

**Figure:**
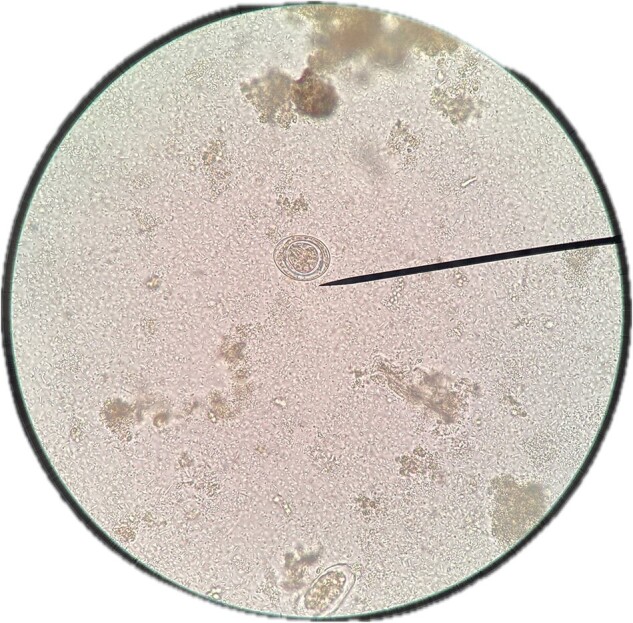

**Disclosures:**

**All Authors**: No reported disclosures

